# *TrexAB*, a novel tetracycline resistance determinant in *Streptococcus dysgalactiae*

**DOI:** 10.3389/fcimb.2025.1583926

**Published:** 2025-06-30

**Authors:** Marte Glambek, Morten Kjos, Marita T. Mårli, Zhian Salehian, Steinar Skrede, Audun Sivertsen, Bård R. Kittang, Oddvar Oppegaard

**Affiliations:** ^1^ Department of Medicine, Haukeland University Hospital, Bergen, Norway; ^2^ Department of Clinical Science, Faculty of Medicine, University of Bergen, Bergen, Norway; ^3^ Faculty of Chemistry, Biotechnology and Food Science, Norwegian University of Life Sciences, Ås, Norway; ^4^ Department of Microbiology, Haukeland University Hospital, Bergen, Norway; ^5^ Department of Internal Medicine, Haraldsplass Deaconess Hospital, Bergen, Norway

**Keywords:** antibiotic resistance, tetracycline, *Streptococcus dysgalactiae*, natural transformation, ABC transporter

## Abstract

**Background:**

*Streptococcus dysgalactiae* (SD) is a potent pathogen associated with infections in a broad range of host species. Notably, a substantial proportion of SD isolates exhibit reduced susceptibility to tetracycline but lack identifiable resistance determinants. In the present study, we wanted to explore the genetic basis for this low-grade resistance to tetracycline.

**Methods:**

Genome-wide association studies were performed on a collection of 407 SD genomes to identify potential novel resistance determinants. Two strains of SD, belonging to each of the subspecies *dysgalactiae* and *equisimilis* were used for mutagenesis. Natural transformation was exploited to knock out resistance gene candidates, and the resultant mutants were compared with their respective wildtypes regarding susceptibility to tetracycline, doxycycline, minocycline, tigecycline, erythromycin, gentamicin, clindamycin and ciprofloxacin.

**Results:**

We identified a two gene operon, herein designated *trexAB*, significantly associated with reduced susceptibility to tetracycline. The proteins encoded by the operon were predicted *in silico* to constitute a heterodimeric efflux transporter. The knockout of *trexAB* led to a 16- to 32-fold reduction in minimum inhibitory concentration (MIC) for tetracycline and a 4-fold reduction in MIC for tigecycline in the investigated strains. No differences between mutants and wildtypes were observed for other antibiotics included in the test panel. Whole genome alignment of mutants and their respective wildtypes revealed no differences other than the expected differences caused by the knockout.

**Conclusion:**

We have characterized a novel operon causing low-grade resistance to tetracycline in SD. The MIC distribution of *trexAB*-positive isolates is intersected by the current EUCAST susceptibility breakpoint, and our findings are relevant for future revisions and determinations of adequate breakpoints for tetracycline in *S. dysgalactiae*.

## Introduction

Tetracyclines were among the first broad-spectrum antibiotics discovered. The limited number of side-effects together with the availability of oral formulations, made tetracyclines attractive choices in both clinical and agricultural settings. The tetracyclines are divided into three different generations, where the first generation comprises tetracycline, oxytetracycline and chlortetracycline, the second generation includes minocycline and doxycycline, and the glycylcycline tigecycline constitutes the third generation (Thaker et al., 2010).

The tetracyclines inhibit bacterial protein synthesis by binding to the bacterial ribosome, disturbing the bacteria’s ability to synthesize proteins. Bacterial resistance against tetracycline occurs by different mechanisms, which mainly fit into the categories of efflux systems, ribosomal protection and drug destruction (Thaker et al., 2010). The more than 60 unique tetracycline resistance determinants characterized to date, indicate that drug efflux is the main resistance strategy in gram-negative bacteria, whereas ribosomal protection is the most common mechanism in gram-positive bacteria (Roberts, 2024). To be defined as a unique resistance gene in this setting, the sequence homology to genes of known function must be lower than 79% at the amino acid level (Levy et al., 1999).

We recently characterized antimicrobial susceptibility patterns of *Streptococcus dysgalactiae* (SD), a gram-positive pathogen known to infect a broad range of host species (Glambek et al., 2024). We explored resistance in a One Health perspective, including both *S. dysgalactiae* subspecies *dysgalactiae* (SDSD) associated with bovine and ovine infections, and *S. dysgalactiae* subspecies *equisimilis* (SDSE) predominantly targeting other animals and humans. Surprisingly, we observed a trimodal distribution of minimum inhibitory concentrations (MIC) values to tetracycline, and a relative high proportion of low-grade tetracycline resistant isolates without an identifiable genetic resistance determinant.

The central cluster of SD isolates had MIC values ranging between 0.5 and 4 μg/ml (herein referred to as the transition zone), and thus encircled the EUCAST breakpoint between sensitive and resistant, suggestive of low-grade phenotypic resistance. Canonical *tet*-genes were identified in nearly all isolates with MIC values above the transition zone, whereas isolates in the central cluster generally did not encode identifiable resistance genes.

In the present study, we explore the underlying mechanism for this low-grade resistance using genome-wide association studies and mutant construction. We report the identification of a novel two-gene operon associated with the low-grade resistance phenotype, likely encoding proteins that together function as an ABC efflux transporter.

## Materials and methods

### Bacterial isolates

A collection of 407 SD strains procured from human and animal associated infections in Norway during 2018–2019 was investigated in this study. The isolates have previously been whole genome sequenced, examined for antibiotic resistance genes and susceptibility tested for tetracycline (Glambek et al., 2024).

In accordance with the phenotypic definition proposed by Vieira et al (Vieira et al., 1998), we defined SDSD *in silico* as genomes harboring the Lancefield group C-antigen operon, lacking the streptolysin S operon (corresponding to an α- or nonhemolytic reaction on blood agar), and lacking the streptokinase gene (inferring that streptokinase activity on human plasminogen does not occur). All other genomes were classified as SDSE (Glambek et al., 2024).

### *In silico* analysis

DBGWAS was used to search for genetic variants associated with low-grade tetracycline resistance (Jaillard et al., 2018). Strains harboring known tetracycline resistance genes were excluded from the analyses, and minimum inhibitory concentration (MIC) level was used as phenotype-indicator, with a MIC phenoThreshold of 0.5 μg/ml. Genetic variants identified by DBGWAS were mapped to genomic location and inspected using the Geneous Prime v 2024.0 software. The predicted function of annotated genes at matching loci was evaluated by screening for conserved functional domains using CD search (Wang et al., 2022) and InterProScan 102.0 (Blum et al., 2024), with default settings. Searches for homologue genes and proteins to our candidate tetracycline resistance genes were done using megaBLASTn and BLASTp, respectively.

A core genome single-nucleotide polymorphism phylogeny was generated by CSI Phylogeny at the Center for Genomic Epidemiology (Kaas et al., 2014) using default settings and the SDSE type strain NCTC13762 as a reference. The resulting maximum likelihood phylogenetic tree was visualized and annotated using the Interactive Tree of Life platform, iTol v6 (Letunic and Bork, 2021).

### Screening of global collection of SD genomes

For comparative analysis, we downloaded a global collection of SD genomes from published epidemiological studies available from GenBank and PubMLST. These included human associated isolates collected in Australia (Xie et al., 2024), Canada (Lother et al., 2017) and Japan (Shinohara et al., 2023), bovine isolates from Canada (Vélez et al., 2017), as well as swine and horse isolates from Italy, US and Portugal (Pinho et al., 2016; Cinthi et al., 2023). The genomes were *de novo* assembled using SPAdes v 5.14 (Bankevich et al., 2012), annotated using RAST v 1.073 (Aziz et al., 2008), and screened for the presence of candidate genes using the Geneious Prime v 2024.0 software.

### Growth conditions and susceptibility testing

In the knockout experiments, bacteria were grown in airtight tubes in C-medium (Lacks and Hotchkiss, 1960) or on brain heart infusion (BHI) agar plates at 37 °C in 10% CO_2_. For selection of knock out mutants, kanamycin was added to the BHI agar to a final concentration of 400 μg/ml.

Knockout mutants and their respective wild types were examined for susceptibility to tetracycline, doxycycline, minocycline, tigecycline, erythromycin, gentamicin, clindamycin and ciprofloxacin according to the NORM protocol (NORM, 2019). Briefly, isolates were plated on Mueller-Hinton agar supplemented with defibrinated horse blood and β-NAD. MIC-levels were determined using MIC-gradient strips.

### Synthetic peptides

Synthetic nature quorum sensing peptide pheromones, XIP1 (aa: EFDWWNLG) and XIP2 (aa: QVDWWRL) were purchased from Thermo Scientific.

### Construction of deletion fragment and deletion of *trexAB* by natural transformation

A linear DNA fragment for homologous recombination to delete *trexAB* was assembled using overlap extension PCR (Higuchi et al., 1988). Amplicons of approximately 2 kb length of flanking sequences to the *trexAB*-operon in addition to a core sequence consisting of the so-called Janus cassette (Sung et al., 2001), encoding a kanamycin resistance cassette and a *rpsL*-allele, were made by PCR and extracted from agarose gel. The *trexAB* upstream and downstream fragments were merged to the 5’ end and 3’ end of the Janus cassette, respectively, making a DNA construct to create genetic knockouts (Higuchi et al., 1988). Primers used are listed in **Table 1**.

**Table 1:** Primers.

Purpose	Primer sequence 5’-3’	Reference
Amplifying upstream fragment	**GAAGACTGAGAAGCCATCAC**	This study
Amplifying upstream fragment	CACATTATCCATTAAAAATCAAAC**GTTATCCTCCTTCTTCTTTTCAG**	This study
Amplifying downstream fragment	GTCCAAAAGCATAAGGAAAG**AATCGTGGCAAGCGTCGTC**	This study
Amplifying downstream fragment	**GCATCTGGTAAGTCCTTTGTC**	This study
Sequence confirmation *trexAB*	**CCCATTAGCATCATGATGGTC**	This study
Sequence confirmation *trexAB*	**TCTGCGACAACAGATTGTCG**	This study
Sequence confirmation *kan* gene	**GTTTGATTTTTAATGGATAATGTG**	Johnsborg et al, 2008
Sequence confirmation *kan* gene	**CTTTCCTTATGCTTTTGGAC**	Johnsborg et al, 2008

Sequence tails not matching target for PCR are marked in grey.

Two SD isolates were selected for functional studies of the *trexAB* operon, the human associated isolate iSDSE_NORM6 of subspecies *equisimilis*, and the bovine associated isolate SDSD24 of subspecies *dysgalactiae*. These strains contained an intact *trexAB* operon and displayed low grade resistance to tetracycline, without possessing any validated tetracycline resistance genes. These strains also possessed a complete and intact apparatus for competence and natural transformation (Mårli et al., 2024).

The natural transformation procedure was adapted from the protocol described by Mårli and co-workers (Mårli et al., 2024). Briefly, overnight cultures of isolates to be transformed were diluted in C-medium to an initial OD_600_ of 0.05 for further incubation until reaching OD_600_ 0.2. The cultures were again diluted to OD_600_ 0.03 and finally grown to OD_600_ 0.05 before approximately 400 ng of DNA-construct and 250 ng of XIP1 (iSDSE_NORM6) or XIP2 (SDSD24) was added to 1 ml culture. Cultures were further incubated at 37°C for 3–4 hours and then plated on BHI agar containing 400 μg/ml kanamycin and grown overnight for selection of kanamycin resistant mutants. Cultures without the added DNA-construct, were used as negative controls.

### Whole genome sequencing

Genomic DNA was purified using MagNA Pure extraction kit (Roche Life Science). Whole genome sequencing of knockout mutant strains was performed at Haukeland University Hospital on an Illumina 4,000 HiSeq system to produce 150 bp paired end reads, as previously described (Oppegaard et al., 2017). The genomes of mutant and wildtype strains were aligned with Mauve, and manually compared for insertion, deletion, and mutation events.

## Results

### Identification of tetracycline resistance determinants

**Figure 1:**
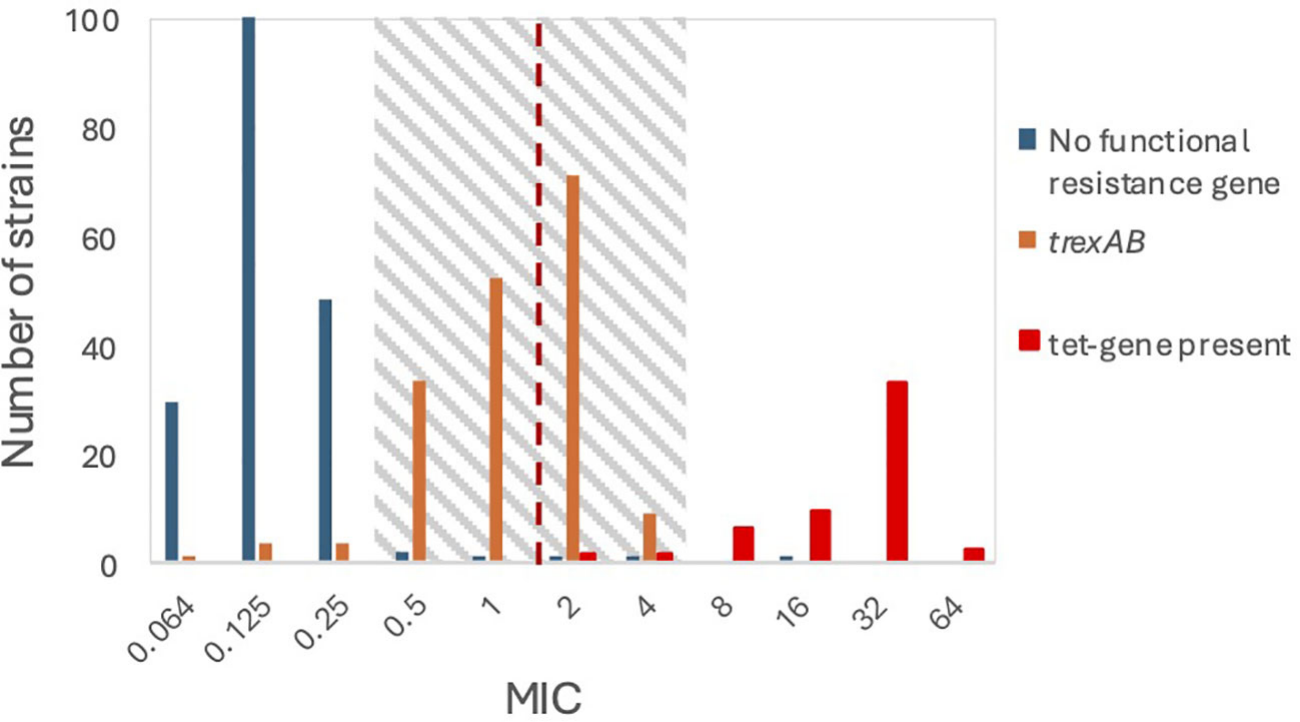
Susceptibility in relation to genes known or presumed to be associated with resistance to tetracycline. The dotted line represents the EUCAST breakpoint between susceptibility and resistance. The MIC transition zone is highlighted with grey shading.

Genome-wide associating studies with DBGWAS identified fifteen genetic regions with at least one polymorphism significantly associated with variations in MIC-level. Only two regions were associated with an increased MIC-level. One of these was a single C/G synonymous mutation in the YidA sugar phosphatase gene, which was deemed unlikely to confer tetracycline resistance. The other was 149 overlapping significant hits constituting a two-gene operon of unknown function. This operon was observed in 167 of the 172 strains with MIC values in the transition zone, but only in 13 of the 184 strains with lower MIC values (**Figure 1**), among which 5 strains contained an intact operon, and the remaining 8 strains had one or both genes truncated (**Supplementary Table**).

We compared the products of the two novel genes to previously characterized tetracycline resistance determinants using BLASTp. A low-level homology to TetA(46) and TetB(46) was detected (55% and 59% pairwise amino acid sequence identity, respectively), a tetracycline efflux pump encoded by a two gene operon in *Streptococcus australis* (Warburton et al., 2013). We thus decided to further explore the potential role in tetracycline resistance of our newly discovered operon, herein designated *trexAB* (Tetracycline Resistance EffluX, gene A and gene B).

### Genetic characterization of the *trexAB* operon

The operon comprised the *trexA* gene (1722 base pairs) and *trexB* gene (1743 base pairs). Both genes showed high interstrain homology within our collection of SD, with nucleotide sequence similarity ranging from 98 to 100%.

**Figure 2:**
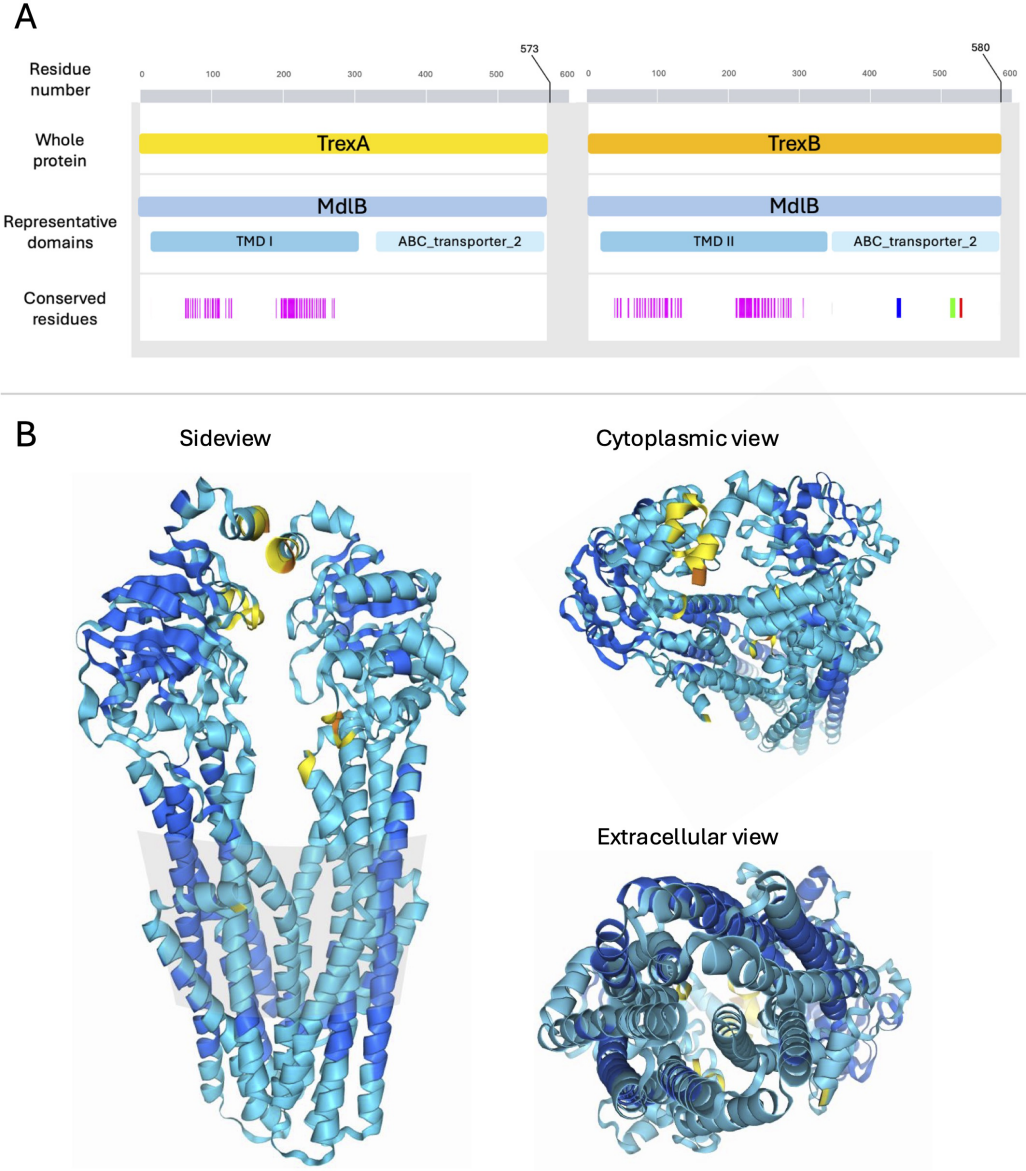
Domains and predicted structure of TrexAB. **(A)** The amino acid sequence of TrexA and TrexB where the numbering of amino acids in the primary sequence is marked in grey scale. Representative domains are highlighted in light blue shades. Conserved residues in TrexA and TrexB determined by InterPro-search representing the heterodimeric interface, the Walker A/P-loop motif, the ABC transporter signature motif, and the Walker B motif are highlighted in pink, blue, green and red, respectively. **(B)** Structures of the heterodimeric TrexAB predicted using Alpha Fold 3. The colors in this model represent per-atom prediction confidence where dark blue, light blue, yellow and orange represent very high, confident, low and very low accuracy, respectively.

Screening the predicted proteins for conserved domains revealed that both TrexA and TrexB harbored MdlB superfamily domains (COG1132), which represent a group of well-characterized ABC-type multidrug transport systems. Typically, these systems are composed of two proteins constituting a dimer spanning the cell membrane, actively exporting toxic substances out of the cell, fueled by the energy generated from hydrolyzing ATP to ADP. In line with this, InterProScan detected the presence of several transmembrane regions gathered in a transmembrane domain (TMD) and an ABC transporter domain constituting the P-loop nucleoside triphosphate hydrolase domain in both TrexA and TrexB (**Figure 2**).

### Genomic context of *trexAB*

**Figure 3:**
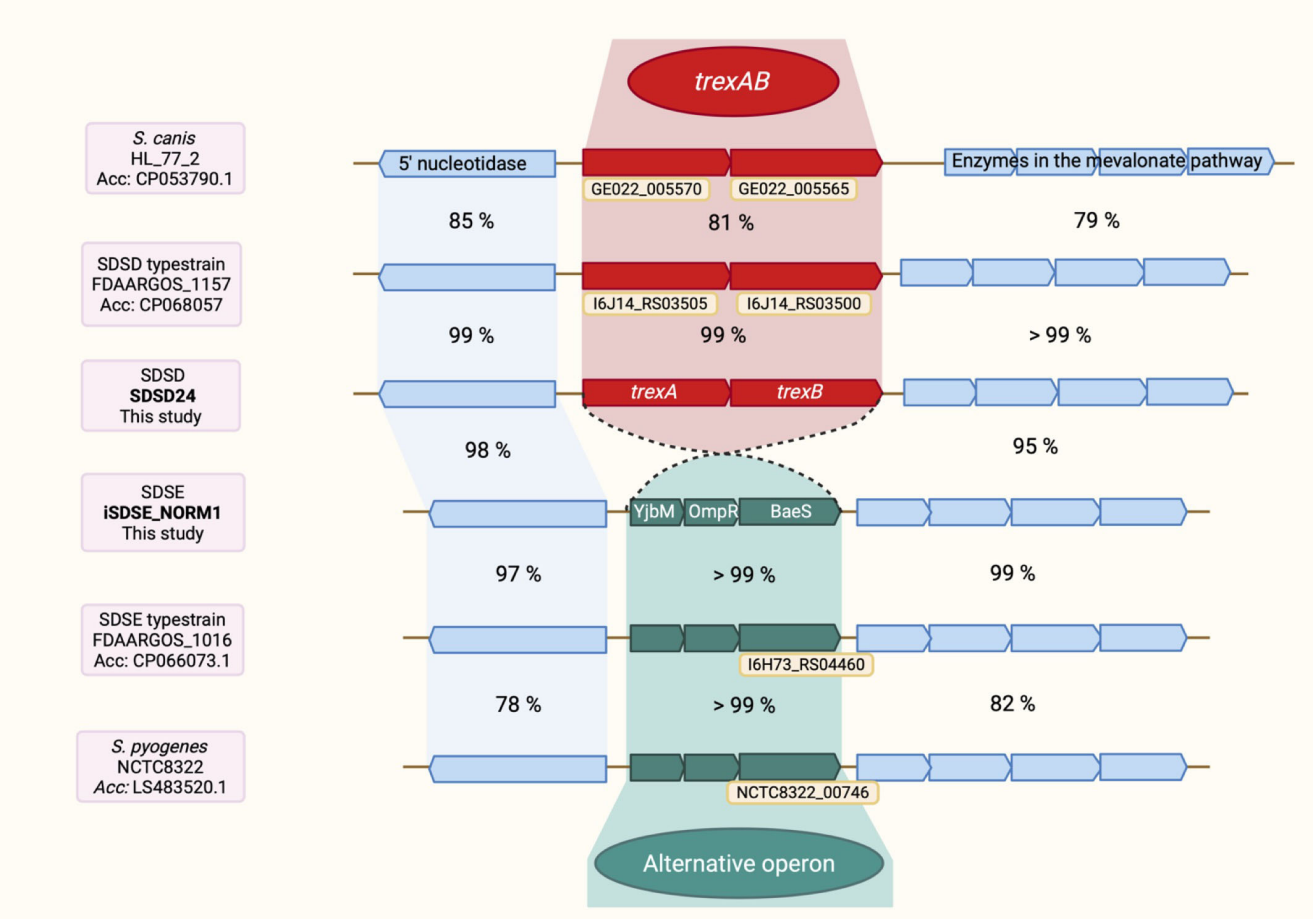
The chromosomal location of *trexAB*. *trexAB* has the closest homolog in *S. canis*, with a sequence identity of 81% at the nucleotide level. The alternative operon is found in *S. pyogenes* in addition to *S. dysgalactiae*. The sequence identity of the alternative operon between *S. pyogenes* and *S. dysgalactiae* is significantly higher than the sequence identity of *trexAB* and its closest homolog in *S. canis*. The close resemblance of the alternative operon in *S. pyogenes* and *S. dysgalactiae* is also in contrast with the flanking regions, which has a much lower sequence identity between the two species. Corresponding ProteinID (to locus tags): “TrexA”: QKG75729.1 (GE022_005570), QQT04366.1 (I6J14_03505); “TrexB”: QKG75728.1 (GE022_005565), QQT04365.1 (I6J14_03500); Alt. operon, 3. gene: QQC56251.1 (I6H73_04460), SRX87322.1 (NCTC8322_00746). Created with Biorender.com, https://app.biorender.com/illustrations/6836d76968fa6ae428cd3cb3.

The *trexAB* operon had a conserved genomic location between an operon containing four genes of the mevalonate pathway and the *s5nA*-nucleotidase gene. In strains lacking *trexAB*, the same genomic location was found to be occupied by a three-gene operon of unknown function (**Figure 3**). The predicted proteins of these three genes all appear to be involved in signal transduction mechanisms, harboring the domains belonging to YjbM superfamily (COG2357), OmpR superfamily (COG0745) and BaeS superfamily (COG0642), respectively. All isolates harbored one of the two operons, and they were mutually exclusive. The genetic region was highly conserved, and we did not find any association between *trexAB* or the alternative operon and known conjugative mobile genetic elements. Differently, IS-elements were found immediately upstream or downstream of *trexAB* in 55 of the isolates.

### Distribution of *trexAB*

**Figure 4:**
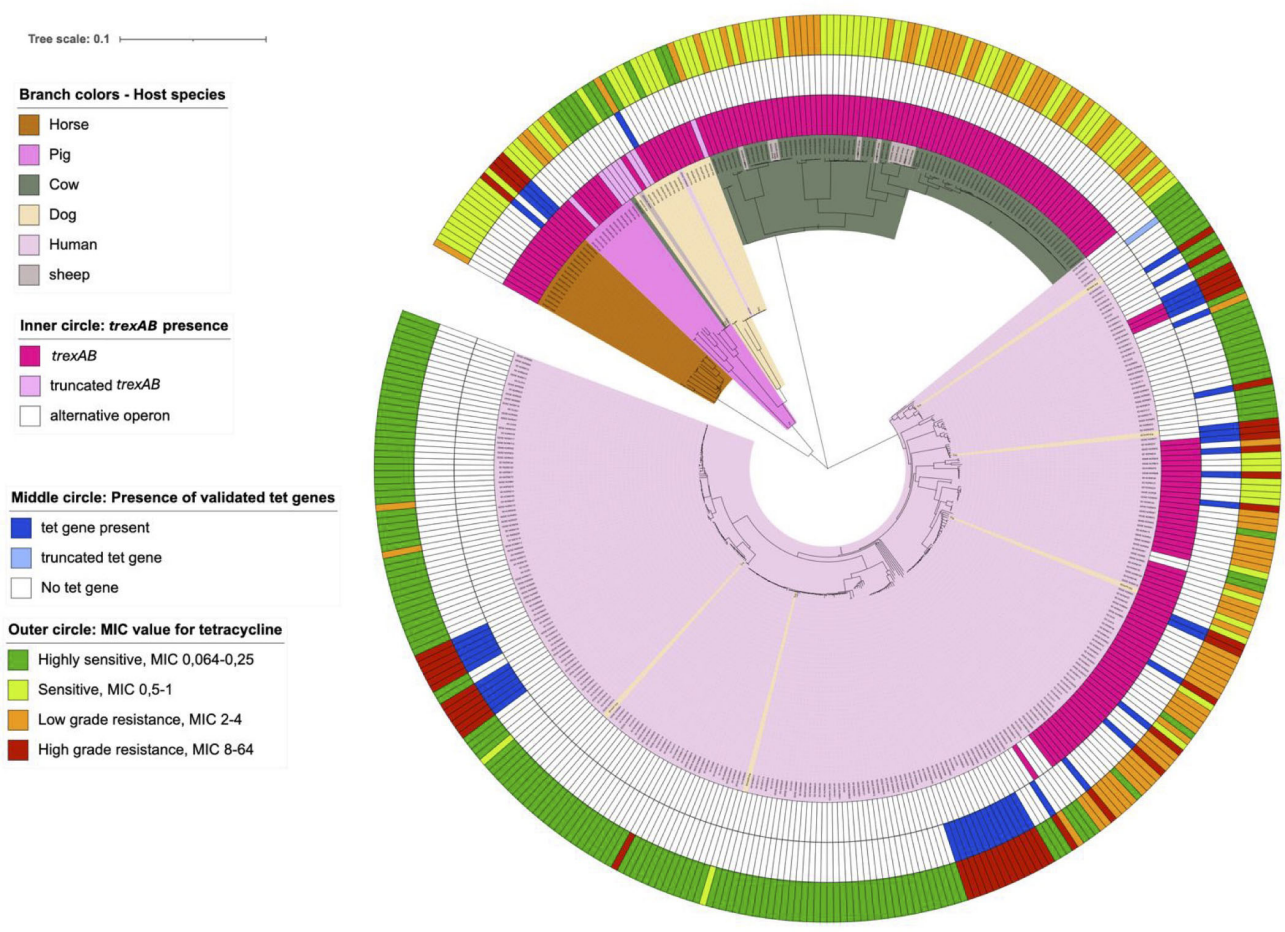
The relationship between genotypic and phenotypic tetracycline resistance and their linkage to phylogenetic distribution. Scale indicates substitutions per site. The phylogenetic tree is constructed based on a core genome single nucleotide polymorphism alignment on maximum likelihood method.

The *trexAB* operon was distributed among SD collected from all ecovars. Notably, all strains (83 SDSD and 2 SDSE) originating from cattle and sheep possessed this operon, as did all isolates (SDSE) from pigs and horses, while only 69 out of 274 isolates (SDSE) associated with humans harbored *trexAB*. The operon was limited to specific phylogenetic clades of human associated SD, predominantly belonging to multilocus sequence type 29 clonal complex. Among the SD isolates (SDSE) procured from dogs, four of 20 isolates were lacking *trexAB*. However, phylogenetically these four isolates clustered with human-associated SD isolates also lacking *trexAB* (**Figure 4**).

Further examination of collections of SD genomes available online revealed results in accordance with the findings regarding our strain collection. We found *trexAB* to be present in 22% (n = 294) isolates originating from Australia, 23% (n = 137) isolates from Japan and 7% (n = 122) isolates from Canada, all isolates associated with human infection or carriage. The *trexAB* carriage of SD isolates from livestock was higher and found in 100% of SD isolates in three different collections of isolates from cattle (n = 86), swine (n = 97) and horses (n = 14), respectively. These animal associated SD were isolated from widespread geographical areas, including locations in China, North America and Europe, confirming a global distribution of *trexAB*.

In BLASTp searches for TrexA and TrexB homologs in other bacterial species, the closest match was found in *Streptococcus canis*, with 100% query coverage and sequence identity of 88% and 89% on the aa level regarding TrexA and TrexB, respectively. Homologs with limited sequence identity (60 – 75%) were also detected in other animal associated streptococcal species, such as *Streptococcus suis*, *Streptococcus phocae* and *Streptococcus iniae*. No homologs were detected among typical human pathogenic species. Interestingly, the only significant homology found to the gene products of the alternative operon was in *Streptococcus pyogenes*, with both query coverage and sequence identity approximating 100% both on the aa and on the nucleotide level (**Figure 3**).

### Knockout of *trexAB*

Successful construct of SDSD24Δ*trexAB* and iSDSE_NORM6Δ*trexAB* knockout mutants was confirmed by whole genome sequencing, and the difference between wild type and mutant was limited to the expected exchange of *trexAB* with the kanamycin cassette. In susceptibility testing for tetracycline, minocycline, tigecycline, doxycycline, erythromycin, gentamicin, clindamycin and ciprofloxacin, both transformant strains showed an increased susceptibility for tetracycline and tigecycline compared to their respective wild types, with a 16- to 32-fold reduction in MIC for tetracycline and a 4-fold reduction in MIC for tigecycline (**Table 2**). For iSDSE_NORM6Δ*trexAB*, a small difference was noted also regarding susceptibility to minocycline, with MIC of 0.032 μg/ml compared to 0.064 μg/ml for the wild type. For all other antibiotics tested, no difference in susceptibility between mutant and wild type was observed.

**Table 2:** MIC values for wildtype and knockout strains.

	MIC for strain iSDSE_NORM6 (*)	MIC for strain iSDSE_NORM6 Δ*trexAB* (*)	MIC for strain SDSD24 (*)	MIC for strain SDSD24Δ*trexAB* (*)
Tetracycline	2 (2)	0.125 (0.125)	4 (4-8)	0.125 (0.125)
Doxycycline	0.125 (0.125-0.25)	0.125 (0,125)	0.25 (0.125-0.25)	0.125 (0.125)
Minocycline	0.064 (0.064)	0.032 (0.032)	0.064 (0.064)	0.064 (0.064)
Tigecycline	0.125 (0.125)	0.032 (0.032)	0.125 (0.125)	0.032 (0.032)
Erythromycin	0.25 (0.25)	0.25 (0.25)	0.25 (0.25)	0.25 (0.25)
Gentamicin	16 (8-16)	8 (8-16)	8 (8)	8 (8)
Clindamycin	0.25 (0.25)	0.25 (0.25)	0.25 (0.25)	0.25 (0.25)
Ciprofloxacin	0.5 (0.5)	0.5 (0.5)	1 (1)	0.5 (0.5-1)

*Range of values for 3 biological replicates.

## Discussion

In the present study, we demonstrate that a two-gene operon designated *trexAB* is associated with reduced susceptibility to tetracycline in SD, and that a targeted knockout of the operon lead to a 16- to 32-fold decrease in tetracycline MIC-values compared to the wildtype strains. The operon has a widespread dissemination and conserved chromosomal location without indications of being located on a mobile genetic element.

Notably, the *trexAB*-operon appears to have a skewed phylogenetic ecological distribution. Whereas only 25% of the human associated isolates in our collection contained the operon, almost all animal-associated isolates were found to carry *trexAB*. A similar distribution is evident in epidemiological collections of SD genomes available in GenBank and PubMLST.

Interestingly, the skewed distribution of *trexAB* between human- and animal-associated isolates is in line with the detection of the closest homolog to *trexAB* in a dog-associated species, *S. canis*, while the alternative operons dissemination is limited to the strictly human pathogen *S. pyogenes*. Combined with the mutual exclusivity of these two operons, this could point to interspecies horizontal genetic exchange and SD evolution occurring within their respective ecological niches. A similar evolutionary phenomenon has also been inferred in several previous genomic studies (Bessen et al., 2005; Ward et al., 2009; Porcellato et al., 2021). Moreover, extensive exchange of genetic material between SD and *S. pyogenes* has previously been documented *in silico* (Xie et al., 2024), as have adaptations of SD to host species through presumed tailored genetic content specific to SD of each host species (Porcellato et al., 2021). Nevertheless, further studies are needed to elucidate the potential origin of these operons.

*In silico* predictions of the domain architecture of the two amino acid sequences encoded in *trexAB*, revealed typical features of a heterodimeric multidrug resistance transporter (MDR) transporter (Lubelski et al., 2004; Matsuo et al., 2007; Torres et al., 2009; Reilman et al., 2014; Hürlimann et al., 2016). These transporters are typically shown capable of exporting a selection of substances across the cell membrane. Susceptibility testing for several antibiotics demonstrated an effect by *trexAB* only for tetracycline and to a lesser extent tigecycline, which provides a tenuous foundation for interpreting *trexAB* as a multidrug transporter. However, we only evaluated *trexAB* in relation to antibiotics, whereas tests of other MDR transporters have included a wider range of noxious substances like ethidium bromide, safranin, doxorubicin, pyrroles and acriflavine (Orelle et al., 2019). Thus, a broader selection of substrates for efflux by *trexAB* than demonstrated here is possible.

The level of resistance to tetracycline caused by *trexAB* seems to be modest, and the impact on susceptibility to tigecycline even more so. As such, the clinical significance of harboring this operon on treatment efficacy is uncertain. Notwithstanding, future vigilance towards potential treatment failures is warranted. Regardless of clinical impact, interpretation of tetracycline resistance in *trexAB*-positive isolates undoubtably represent a challenge, as the MIC distribution of this population is intersected by the current EUCAST breakpoint. Due to inherent technical and analytical variability in susceptibility testing, such isolates will thus be interchangeably classified as resistant or susceptible to tetracycline. Notably, in 2023 EUCAST removed the category “Susceptible, increased exposure” for tetracycline in beta-hemolytic streptococcal species, including SD. They argued that a pharmacodynamic and pharmacokinetic rationale for an intermediate category was not evident, and lowered the breakpoint for resistance from “above 2 μg/ml” to “above 1 μg/ml”. Considering the widespread distribution of low-grade resistant *trexAB*-positive SD isolates, the implications for dosage and clinical efficacy need to be carefully evaluated before further revising the EUCAST breakpoints.

A limitation of the knockout experiments in this study is the fact that both resistance genes were removed in one maneuver, making it difficult to decipher the individual contribution of TrexA and TrexB. However, several others have documented the need for the contribution from both half transporters for the function of a heterodimeric efflux pump (Matsuo et al., 2007; Garvey et al., 2010; Warburton et al., 2013). In addition, our collection contained isolates where *trexA* alone was truncated, which in each case was associated with full susceptibility to tetracycline.

Another potential limitation is the use of MIC-strips for susceptibility testing, as broth microdilution or disc diffusion are the reference methods proposed by EUCAST (EUCAST, 2025). Nevertheless, a distribution of tetracycline susceptibility encircling the current breakpoint is evident also in studies of SD using broth microdilution methodology (McDougall et al., 2014; Jensen et al., 2024). Moreover, we have disc diffusion data for one third of the isolates in the present study, and the susceptibility distribution is congruent with the MIC-strip results (data not shown).

In conclusion, we have investigated the cause for low grade tetracycline resistance in *S. dysgalactiae* and found the underlying genetic factor to be the two gene operon *trexAB* encoding a hitherto uncharacterized ABC transporter. The tetracycline MIC distribution of the *trexAB*-positive isolates is intersected by the current EUCAST breakpoint, and the clinical implications of this should be subject to scrutiny.

## Data Availability

The datasets presented in this study can be found in online repositories. The names of the repository/repositories and accession number(s) can be found in the article/**Supplementary Material**.
